# In vitro cytotoxic and in silico activity of piperine isolated from *Piper nigrum* fruits Linn

**DOI:** 10.1186/s40203-015-0013-2

**Published:** 2015-10-29

**Authors:** Padmaa M. Paarakh, Dileep Chandra Sreeram, Shruthi S. D, Sujan P. S. Ganapathy

**Affiliations:** Department of Pharmacognosy, The Oxford College of Pharmacy, 6/9, I Cross, Begur Road, Hongasandra, Bangalore, 560068 Karnataka India; R & D [Phytochemistry], Natural Remedies Private limited, Bangalore, India; Microbiology and Cell Biology Department, Indian Institute of Science, Bangalore, Karnataka India; Research and Development Centre, Olive LifeSciences Pvt. Ltd., Nelamangala, Bangalore, 562123 Karnataka India

**Keywords:** *In vitro* cytotoxic activity, In silico docking studies, Isolation, Piperine, *Piper nigrum*

## Abstract

**Background:**

*Piper nigrum* [Piperaceae], commonly known as black pepper is used as medicine fairly throughout the greater part of India and as a spice globally.

**Purpose:**

To isolate piperine and evaluate *in vitro* cytotoxic [antiproliferative] activity and in silico method.

**Methods:**

Piperine was isolated from the fruits of *P.nigrum*. Piperine was characterized by UV,IR, ^1^H-NMR, ^13^C-NMR and Mass spectrum. Standardization of piperine was done also by HPTLC fingerprinting. *In vitro* cytotoxic activity was done using HeLa cell lines by MTT assay at different concentrations ranging from 20 to 100 μg/ml in triplicate and in silico docking studies using enzyme EGFR tyrosine kinase.

**Results:**

Fingerprinting of isolated piperine were done by HPTLC method. The IC_50_ value was found to be 61.94 ± 0.054 μg/ml in *in vitro* cytotoxic activity in HeLa Cell lines. Piperine was subjected to molecular docking studies for the inhibition of the enzyme EGFR tyrosine kinase, which is one of the targets for inhibition of cancer cells. It has shown −7.6 kJ mol^−1^ binding and 7.06 kJ mol^−1^ docking energy with two hydrogen bonds.

**Conclusion:**

piperine has shown to possess *in vitro* cytotoxic activity and in silico studies.

## Background

Cancer is one of the highest impacting diseases worldwide with significant morbidity and mortality rates. The current known therapies are based on radio and chemotherapies and although in many cases, the patients have their health re-established, the treatment is very painful since their immunological system is severely compromised, because these procedures are not cells selective [Leticia et al. 2013]. Substantial advances have been made in understanding the key roles of receptor tyrosine kinase (RTK) in the signalling pathways that govern fundamental cellular processes, such as proliferation, migration,metabolism, differentiation and survival. In the normal cells RTK activity is tightly controlled. When they are mutated or structurally altered, they become potent oncoproteins which leads to abnormal activation of RTKs in transformed cells has been shown to be causally involved in the development and progression of many human cancers (Andreas et al. 2004; Wajapeyee et al. 2006).

Tyrosine kinases are an especially important target because they play an important role in the modulation of growth factor signaling. There are multiple types of targeted therapies available, including monoclonal antibodies, inhibitors of tyrosine kinases and antisense inhibitors of growth factor receptors. But we have focussed only on inhibitors of receptor tyrosine kinases. Tyrosine kinases play a critical role in the modulation of growth factor signaling. Activated forms of these enzymes can cause increases in tumor cell proliferation and growth, induce antiapoptotic effects and promote angiogenesis and metastasis. In addition to activation by growth factors, protein kinase activation by somatic mutation is a common mechanism of tumor genesis. Ligand binding induces dimerization of these receptor tyrosine kinases, resulting in autophosphorylation of their cytoplasmic domains and activation of tyrosine kinase activity. Multiple cytoplasmic signaling pathways, including the Ras/Raf mitogen-activated protein kinase pathway, the phosphoinositol 3’-kinase/Akt pathway, the signal transducer and activator of transcription 3 pathway, the protein kinase C pathway, and scaffolding proteins may then be activated (Schlessinger 2000; Bogdan and Klambt 2001). Intracellular mediators in these pathways transduce signals from membrane receptors through the cytosol and into the nucleus, culminating in altered DNA synthesis and cell division as well as effects on a variety of biological processes, including cell growth, migration, differentiation and death (Carpenter and Cohen 1990; Blume-Jensen and Hunter 2001). Because all of these effects are initiated by receptor tyrosine kinase activation, they are key targets for inhibitors.

The tyrosine kinase inhibitors compete with the ATP binding site of the catalytic domain of several oncogenic tyrosine kinases. They are orally active, small molecules that have a favorable safety profile and can be easily combined with other forms of chemotherapy or radiation therapy. Several tyrosine kinase inhibitors (TKIs) have been found to have effective antitumor activity and have been approved or are in clinical trials. The inhibitors used are imatinib mesylate, gefitinib, erlotinib, lapatinib, canertinib, semaxinib, vatalanib, sorafenib,sutent and leflunomide. TKIs are thus an important new class of targeted therapy that interfere with specific cell signaling pathways and thus allow target-specific therapy for selected malignancies. Use of these targeted therapies is not without limitations such as the development of resistance and the lack of tumor response in the general population. The availability of newer inhibitors and improved patient selection will help overcome these problems in the future (Finley 2003).

The cost of treatment is very high and with lot of side effects. In order to find new natural sources that are biologically active substances from plants have acquired immense attention. A number of studies have been carried out on various plants, vegetables and fruits because they are rich sources of phytoconstituents which prevent free radical damage thereby reducing risk of chronic diseases viz., cancer, cardiovascular diseases etc. This beneficial role of plants has led to increase in the search for newer plant based sources for the treatment of diseases like cancer. One such plant is *Piper nigrum*.

*Piper nigrum*, commonly known as black pepper is used as medicine fairly throughout the greater part of India and considered as King of spice. The plant is reported to possess antiapoptotic, antibacterial, anti-colon toxin, antidepressant, antifungal, antidiarrhoeal, anti-inflammatory, antimutagenic, anti-metastatic activity, antioxidative, antispasmodic, antispermatogenic, antitumor, antithyroid, gastric ailments, hepatoprotective, insecticidal activity, intermittent fever, larvicidal activity, protection against diabetes induced oxidative stress, analgesic,anti-inflammatory, anticonvulsant, antimalarial, antifiliarial, and antifertility activities (Ahmad et al. 2012).

The chemopreventive effects of piperine against several kinds of carcinogen, such as benzo(α)pyrene and 7,12-dimethyl benz(α)anthracene, show its potential as a cancer preventive agent. Administration of piperine (50 mg/kg or 100 mg/kg per day for 7 days) inhibits solid tumor development in mice transplanted with sarcoma 180 cells. A recent study has shown that piperine inhibits breast stem cell self-renewal and does not cause toxicity to differentiated cells. It has been demonstrated that piperine induced apoptosis and increased the percentage of cells in G 2/M phase in 4 T1 cells and induced K562 cells to differentiate into macrophages/monocytes. Piperine also has very good antimetastatic properties against lung metastasis induced by B16F-10 melanoma cells in mice (200 μM/kg) and suppresses phorbol-12-myristate-13-acetate (PMA)-induced tumor cell invasion (Lu et al. 2012).

Piperine is a potent inhibitor of NF-κB, c-Fos, cAMP response element-binding (CREB), activated transcription factor 2 (ATF-2), among others. It suppresses PMA-induced MMP-9 expression via the inhibition of PKCα/extracellular signal-regulated kinase (ERK) 1/2 and reduction of NF-κB/AP-1 activation. Remarkably, piperine also inhibits the functions of P-glycoprotein (P-gp) and CYP3A4, which not only affects drug metabolism but also re-sensitizes multidrug resistant (MDR) cancer cells. Piperine increases the therapeutic efficacy of docetaxel in a xenograft model without inducing more adverse effects on the treated mice by inhibiting CYP3A4, one of the main metabolizing enzymes of docetaxel. The tyrosine kinases inhibitor activity of piperine is not studied till date. The aim of the present study is to isolate piperine from dried fruits of *Piper nigrum* and perform in silico activity and *in vitro* MTT assay to prove its cytotoxic activity.

## Methods

### Plant material

The dried fruits of *Piper nigrum* (Piperaceae) were collected, identified and authenticated by Dr Shiddamallayya N at National Ayurveda Dietetics Research Institute, Bengaluru, Karnataka. A voucher specimen was deposited in the Herbarium of Department of Pharmacognosy, The Oxford College of Pharmacy, Bangalore. The fruits were dried under normal environmental conditions. The dried fruits were powdered and stored in a closed container for further use.

### Drugs and chemicals

DMEM medium (GIBCO), heat-inactivated fetal bovine serum (FBS), trypsin, ethylene-diaminetetraacetic acid (EDTA),PBS and MTT were purchased from Hi media and Sigma Chemicals. All chemicals and reagents used in this study were at least of analytical grade.

### Extraction and isolation procedure

The dried fruits of *P.nigrum* (150 g) was macerated with glacial acetic acid (6 × 300 ml) for 5 min each time. Filter and pooled acetic acid layer was mixed with equal volume of water. Extract with chloroform 3 times and combined chloroform was washed with 10 % sodium carbonate and water. Chloroform layer was dried over anhydrous sodium sulphate and concentrated to dryness at 60 °C. The residue was dissolved in minimum quantity of chloroform,add diethyl ether which resulted in separation of needle shaped crystals of crude piperine. The crude needles were repeatedly crystallized as above to give shinning yellow needles of piperine (0.35 g).

### Characterization of piperine

The structure of Piperine was characterized by UV, IR, NMR and Mass spectrum. HPTLC fingerprinting was done to confirm the presence and purity of Piperine.

### Chromatographic finger printing of the dried fruits of *P. nigrum* using piperine

Weigh 2 g of coarsely powdered drug and transfer to a 250-ml conical flask. Extract with 50 ml of methanol by refluxing for about 20 min and filter. Repeat the process 4–5 times till the raw material is completely exhausted or till the extract is colourless. Combine the extracts and concentrate to a volume of about 100 ml, cool to room temperature. Use the solution for TLC profiling. Standard solution was prepared by dissolving 10 mg of Piperine in 100 ml of methanol. Solvent system used was Hexane: Ethyl acetate (5:3). Apply 20 μl of test solution and 5 μl of standard solution separately on a precoated silica gel 60F_254_TLC plate (E. Merck) of uniform thickness (0.2 mm). Develop the plate in the solvent system till the solvent rises to a distance of 8 cm. Visualization was done after spraying with anisaldehyde-sulphuric acid reagent and followed by heating at 105 °C for 5–10 min. The R_f_ value and colour of the resolved bands were noted.

###  *In vitro* cytotoxic activity using HeLA cell lines by MTT assay

#### Cell culture

HeLa cell line was maintained in DMEM medium (GIBCO) supplemented with 10 % (v/v) heat-inactivated fetal bovine serum (FBS) and 1 % antibiotic solution (penicillin 100U/ml and streptomycin 100 μg/ml) at 37 °C in a humidified atmosphere of 95 % air/5 % CO_2_. The medium was changed every second day, and cells were subcultured when confluency reach to 95 % by 0.25 % trypsin containing 0.02 % ethylene-diaminetetraacetic acid (EDTA) in PBS for 3 min at 37 °C.

#### MTT Assay

The MTT assay was carried out as described previously to measure cell viability (Rahman et al. 2011). Ten thousand cells in 100 μl of DMEM media were seeded in the wells of a 96-well plate. After 24 h, existing media was removed and 100 μl of various concentrations of compound [20–100 μg/ml] were added and incubated for 48 h at 37 °C in a CO_2_ incubator. Control cells were supplemented with 0.05 % DMSO vehicle. At the 48th hour of incubation, MTT (3-(4,5-dimethylthaizol-2-yl)-2,5-diphenyltetrazolium bromide- supplied from Sigma, 10 μl of 5 mg/ml) was added to the plate. The contents of the plate were pipetted out carefully, the formazan crystals formed were dissolved in 100 μl of DMSO, and the absorbance was measured at 550 nm in a microplate reader (Tecan, Infinite F200 Pro). Experiments were performed in triplicate [3 times x 3 wells each time/group] and the results were expressed as mean of percentage inhibition. A graph of the concentration versus percentage growth inhibition was plotted, and the concentration at which 50 % cell death occurred was considered as the IC_50_ value. Before adding MTT, bright field images (Olympus 1 × 81, cellSens Dimension software) were taken for visualizing the cell death.

### In silico activity: molecular docking studies

The three dimensional structure of target protein EGFR tyrosine kinase (PDB ID:2J5F) was downloaded from PDB (www.rcsb.org/pdb) structural database. This file was then opened in SPDB viewer edited by removing the heteroatoms, adding C terminal oxygen. The active pockets on target protein molecule were found out using CASTp server (Binkowski et al. 2003). The ligands were drawn using ChemDraw Ultra 6.0 and assigned with proper 2D orientation (ChemOffice package). 3D coordinates were prepared using PRODRG server (Ghose and Crippen 1987). Autodock V3.0 was used to perform Automated Molecular Docking in AMD Athlon (TM) 2 × 2 215 at 2.70 GHz, with 1.75 GB of RAM. AutoDock 3.0 was compiled and run under Microsoft Windows XP service pack 3. For docking, grid map is required in AutoDock, the size of the grid box was set at 102, 126 and 118 Å (R, G, and B) and grid center −58.865, −8.115, −24.556 for x, y and z-coordinates. All torsions were allowed to rotate during docking. The Lamarckian genetic algorithm and the pseudo-Solis and Wets methods were applied for minimization, using default parameters (Morris et al. 1998).

## Results and Discussions

### The characterization of the piperine (Fig. 1)

**Fig. 1:**
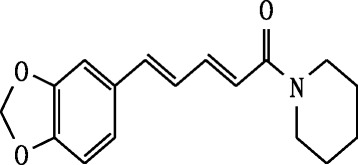
Strucuture of the compound piperine

#### Physical data

Piperine is orange needle shaped crystals, mp 132 °C; lit mp 131–132 °C. Soluble in chloroform and methanol; insoluble in water.

Molecular formula: C_17_H_19_NO_3;_ Molecular weight: 285.342.

#### Spectral data

UV-VIS spectrum shows absorption at 340 nm. The UV-VIS spectrum indicates the presence of chromophoric system in the molecule. The IR spectrum showed shows peaks corresponding to the functional groups present in piperine. ^1^H NMR spectrum (DMSO-d_6_-300 MHz): The signal at δ 1.55–1.71 (6H, m), 3.52 (2H, s), 3.63 (2H, s), 5.98 (2H,s,methylene dioxy-H), 6.44 (1H, d), 6.76 (1H, dd), 6.77 (1H,d), 6.89 (1H, dd), 6.98 (1H, d, aromatic-H), 7.40 (1H, ddd). The δ values were comparable with that of reported ^1^H NMR piperine (Sakpakdeejaroen and Arunporn 2009).

^13^C NMR spectrum (CD_3_OD −300 MHz): the signals at δ 24.66, 25.66,26.67, 43.25, 46.93, 101.24, 105.71,108.46,120.15,122.41,125.41,131.07,138.14,142.39,148.11,148.21 and 165.43. The δ values were comparable with that of reported ^13^C NMR piperine (Avijit et al. 1984).

**Fig. 2:**
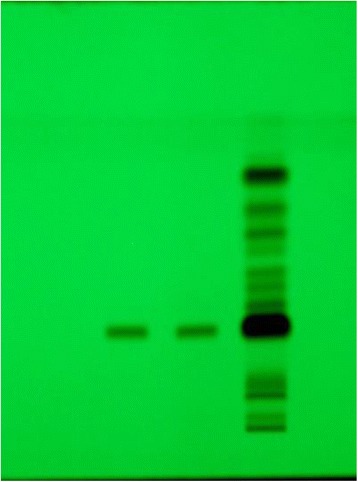
HPTLC profile of standard and isolated piperine and extract of *Piper nigrum* dried fruits at 254 nm. 1: Piperine standard; 2: Isolated Piperine; 3: *P. nigrum* extract

**Fig. 3:**
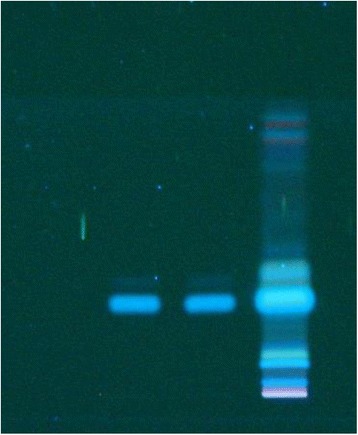
HPTLC profile of standard and isolated piperine and extract of *Piper nigrum* dried fruits at 366 nm. 1: Piperine standard; 2: Isolated Piperine; 3: *P. nigrum* extract

**Fig. 4:**
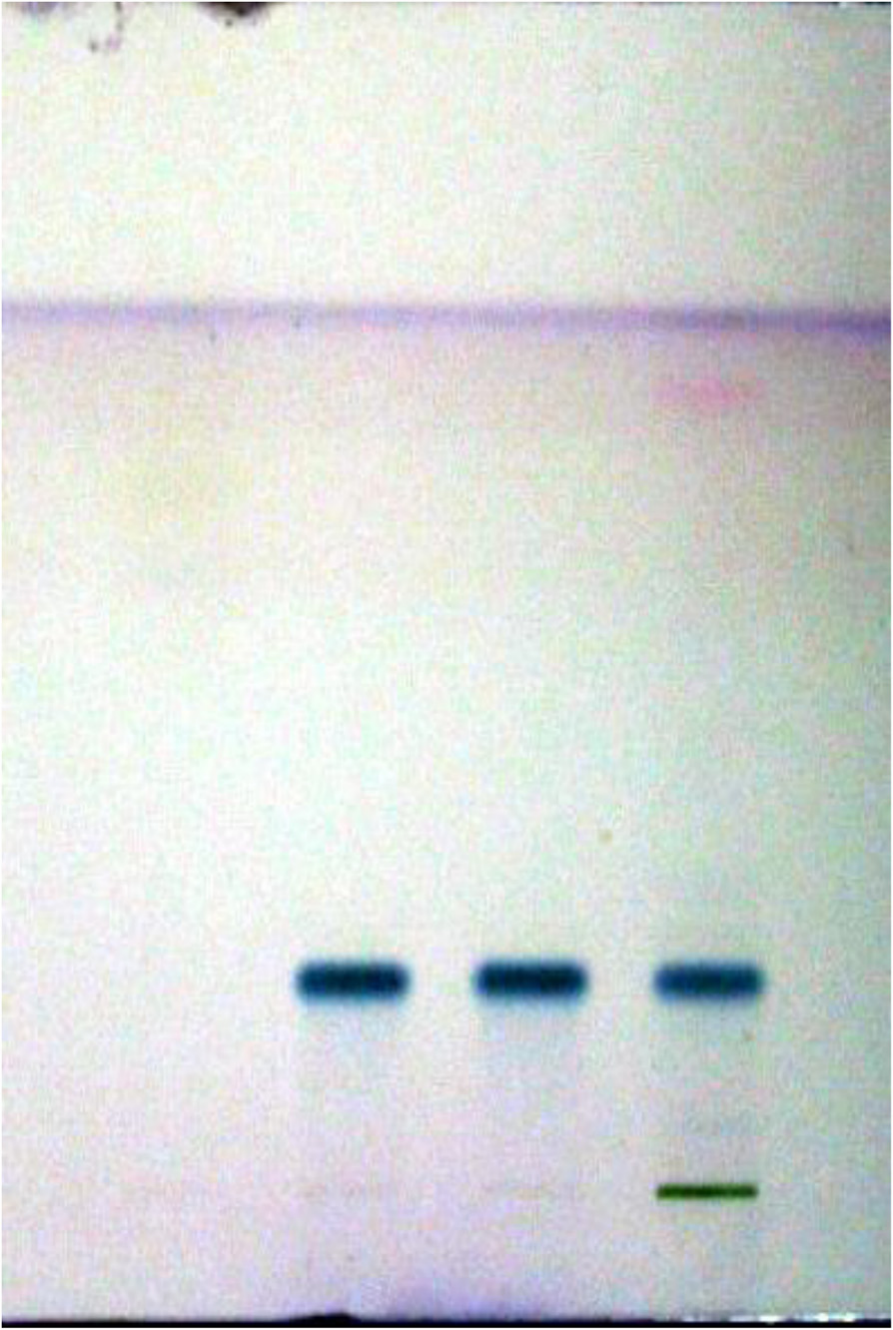
HPTLC profile of standard and isolated piperine and extract of *Piper nigrum* dried fruits after dervatization. 1: Piperine standard; 2: Isolated Piperine; 3: *P. nigrum* extract

The HPTLC fingerprinting confirmed the presence of Piperine (Figs. 2, 3 and 4). A grey coloured band was observed at (R_f_ 0.37) corresponding to piperine is visible in both the test and standard solution tracks under UV at 254 nm,366 nm and after derivatization.

### *In vitro* cytotoxic activity on HeLa cell lines

**Fig. 5:**
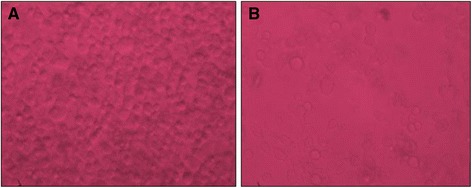
Cytotoxic activity of piperine showing cell death, **a**-control; **b**-treated

The MTT values obtained demonstrated that piperine has cytotoxic effect as the IC_50_ value was found to be 61.94 ± 0.054 μg/ml. Microscopy images representing the cell death caused by the compounds are as seen in Fig. 5. It is very clear that it is cytotoxic agent when compared to control cell with vehicle alone.

### In silico molecular docking studies

The tyrosine kinase receptors have multidomain extracellular Ligands for specific Ligand, a signal pass transmembrane hydrophobic helix and tyrosine kinase domain. The receptor tyrosine kinases are not only cell surfaces transmembrane receptors, but are also enzymes having kinase activity (Bari et al. 2012). In cancer, angiogenesis is an important step in which new capillaries develop for supplying a vasculature to provide nutrient and removing waste material. So tyrosine kinase inhibitor as an anti-angiogenic agent is new cancer therapy. Developing natural drugs and prodrugs as inhibitor is today’s trend. Low molecular weight substances, which inhibit tyrosine kinase phosphorylation block signaling pathway, initiated in the extracellular part of receptor (Paul and Manlay 2002). Since, the type I receptor tyrosine kinase is a major regulator of several distinct and diverse cellular pathways we have evaluated it as a target.

Piperine was taken and docked to get the best conformer. Results were compared for the binding energy, docking energy and number of hydrogen bonds formed. According to the docking results (Table 1), it has binding energy of −7.6 kJ mol^−1^ with two hydrogen bonds formed.

**Table 1:** Molecular docking results of piperine with EGFR tyrosine kinase

Molecule	Binding energy	Docking energy	Inhibitory constant	Intermol energy	H-bonds	Bonding
PR	−7.6	7.06	2.69e-006	−8.22	2	PR::DRG:OAD:TK:A:PRO699:O
						PR::DRG:OAB:TK:A:ARG831:HH12

**Fig. 6:**
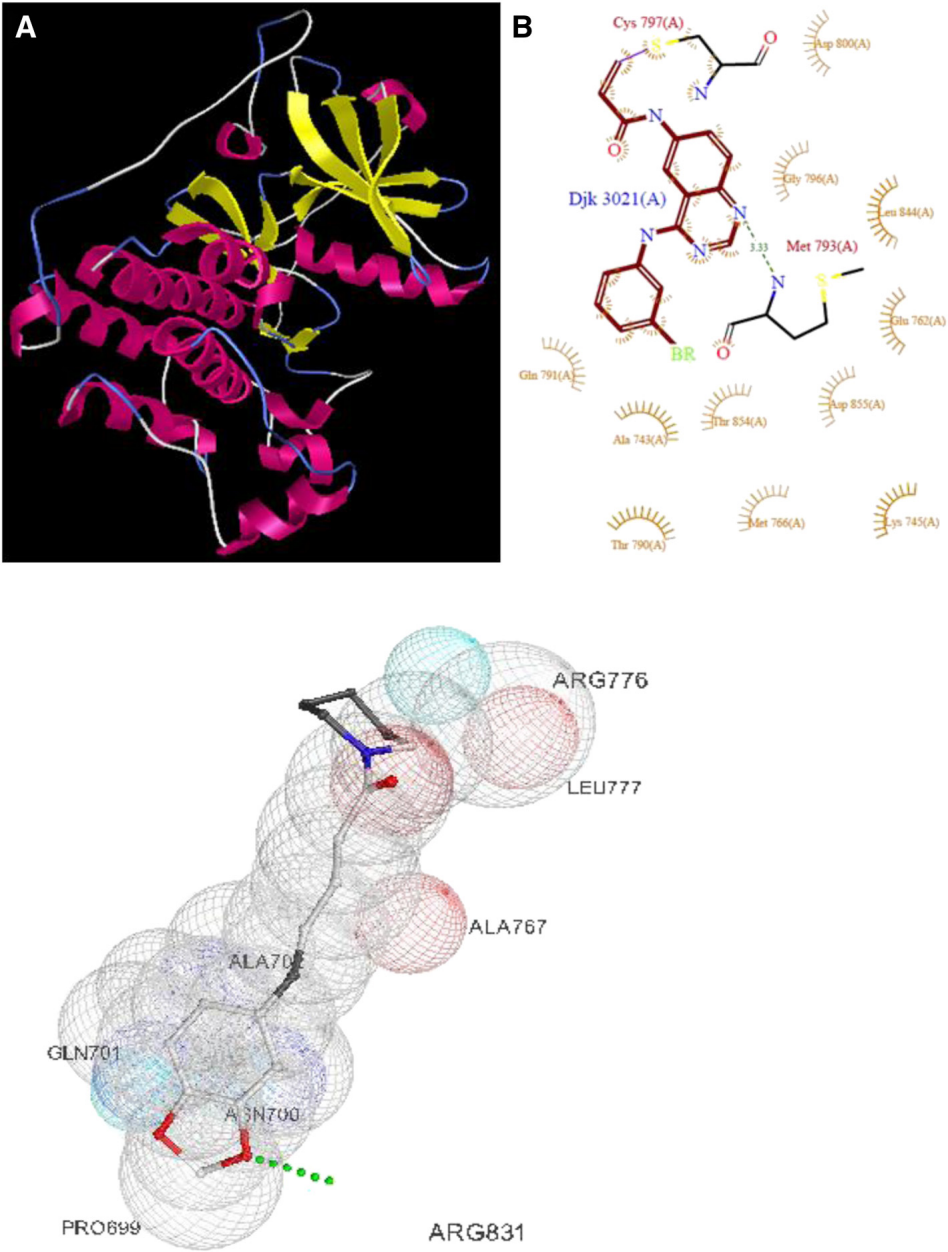
3D structure of EGFR tyrosine kinase from PDB (**a**); Interacting amino acids as predicted from the ligplot (**b**); Enfolding of piperine in the active pocket (**c**)

Molecular docking with EGFR tyrosine kinase domain revealed that, our compound has inhibitory capability and thereby exhibiting interactions with one or the other amino acids in the active pockets as shown in Fig. 6. The topology of the active site of EGFR tyrosine kinase was similar in all synthesized molecules, which is lined by interacting amino acids as predicted from the ligplot (Fig. 6). In * in vitro* studies the molecule emerged to be active against the cell line used in inhibiting the cell growth.

## Conclusion

Piperine has shown to possess * in vitro* cytotoxic activity and in silico studies. The IC_50_ value was found to be 61.94 ± 0.054 μg/ml and in silico studies, it has more number of hydrogen bonds with minimum binding and docking energy and may be considered as inhibitor of EGFR tyrosine kinase. More experiments are required to understand the exact mechanism by which the cells are affected. It is important to correlate the structure of these compounds with their biological effect, which will be valuable to propose new lead compounds with better cytotoxic potential.
